# A novel action mechanism for MPT0G013, a derivative of arylsulfonamide, inhibits tumor angiogenesis through up-regulation of TIMP3 expression

**DOI:** 10.18632/oncotarget.2451

**Published:** 2014-09-08

**Authors:** Chih-Ya Wang, Jing-Ping Liou, An-Chi Tsai, Mei-Jung Lai, Yi-Min Liu, Hsueh-Yun Lee, Jing-Chi Wang, Shiow-Lin Pan, Che-Ming Teng

**Affiliations:** ^1^ Pharmacological Institute, College of Medicine, National Taiwan University, Taipei, Taiwan; ^2^ School of Pharmacy, College of Pharmacy, Taipei Medical University, Taipei, Taiwan; ^3^ The Ph.D. program for Cancer Biology and Drug Discovery, College of Medical Science and Technology, Taipei Medical University, Taipei, Taiwan

**Keywords:** MPT0G013, angiogenesis, TIMP3, proliferation, tumor

## Abstract

Tissue inhibitors of metalloproteinases 3 (TIMP3) were originally characterized as inhibitors of matrix metalloproteinases (MMPs), acting as potent antiangiogenic proteins. In this study, we demonstrated that the arylsulfonamide derivative MPT0G013 has potent antiangiogenic activities *in vitro* and *in vivo* via inducing TIMP3 expression. Treatments with MPT0G013 significantly inhibited endothelial cell functions, such as cell proliferation, migration, and tube formation, as well as induced p21 and cell cycle arrest at the G0/G1 phase. Subsequent microarray analysis showed significant induction of *TIMP3* gene expression by MPT0G013, and siRNA-mediated blockage of TIMP3 up-regulation abrogated the antiangiogenic activities of MPT0G013 and prevented inhibition of p-AKT and p-ERK proteins. Importantly, MPT0G013 exhibited antiangiogenic activities in *in vivo* Matrigel plug assays, inhibited tumor growth and up-regulated TIMP3 and p21 proteins in HCT116 mouse xenograft models. These data suggest potential therapeutic application of MPT0G013 for angiogenesis-related diseases such as cancer.

## INTRODUCTION

The importance of blood vessel growth, or angiogenesis, has been demonstrated as an essential step during embryonic development, tissue repair, and vascular diseases [1]. During recent decades, early pioneers of angiogenic research discovered that growth and metastasis of tumors depend on vascularization. Angiogenic proteins, such as vascular endothelial growth factor-A (VEGF-A) and basic fibroblast growth factor (bFGF), are secreted by tumors to initiate angiogenesis by stimulating proteolytic degradation of capillary basement membranes, thereby promoting endothelial cell migration, proliferation, and formation of tube-like structures [2]. Therefore, angiogenesis is a promising target for anticancer treatments [3].

Previous studies show that the initial stages of angiogenesis involve degradation of extracellular matrices by proteins of the matrix metalloproteinase (MMP) family. Thus, MMPs and MMP inhibitors play important roles in tumor invasion, metastasis, and angiogenesis [4, 5]. The tissue inhibitor of metalloproteinase-3 (TIMP3) is a member of the TIMP family of proteins, which are expressed ubiquitously as natural MMP protease inhibitors. Unlike other TIMP family members, TIMP3 is a secretory protein that binds extracellular matrices [6]. TIMP3 is produced constitutively in the eye by choroidal endothelial cells and retina pigment epithelium (RPE) [7, 8]. Due to its potent MMP-inhibitory activity, TIMP3 affects physiological tissue remodeling and developmental processes by regulating cell growth, invasion, and migration [9]. Under pathological conditions, TIMP3 has been demonstrated as a potent inhibitor of angiogenesis and tumor growth [10]. Thus, silencing of TIMP3 is a common event in solid tumors and promotes tumorigenesis and metastasis by increasing the activity of MMPs. [11]. Mutation of TIMP3 is associated with Sorsby's fundus dystrophy, a macular degenerative disease with submacular choroidal neovascularization [12-14]. Recent studies have demonstrated that TIMP3 inhibits angiogenesis by inhibiting VEGF–KDR binding and downstream signaling pathways, including activation of Akt and ERK. Because TIMP1, TIMP2, and other MMP inhibitors do not inhibit interactions between VEGF and KDR, this effect may be independent of the MMP-inhibitory activity of TIMP3 [15].

In the present study, we investigated antiangiogenic activities of the arylsulfonamide derivative MPT0G013 (3-[1-(3,4-dimethoxy-benzenesulfonyl)-1H-indol-5-yl]-N-hydroxy-acrylamide) *in vivo* and *in vitro*. MPT0G013 was discovered in previous studies, which described potent inhibition of histone deacetylase (HDAC) *in vitro* [16]. However, the effects of this compound on tumor angiogenesis have not been investigated previously. The present data show that MPT0G013 inhibits angiogenesis by up-regulating TIMP3 gene expression in endothelial and tumor cells, indicating the potential of MPT0G013 as a therapeutic agent with dual activities against tumor growth and angiogenesis.

## RESULTS

### MPT0G013 inhibits angiogenesis *in vitro*

**Figure 1:**
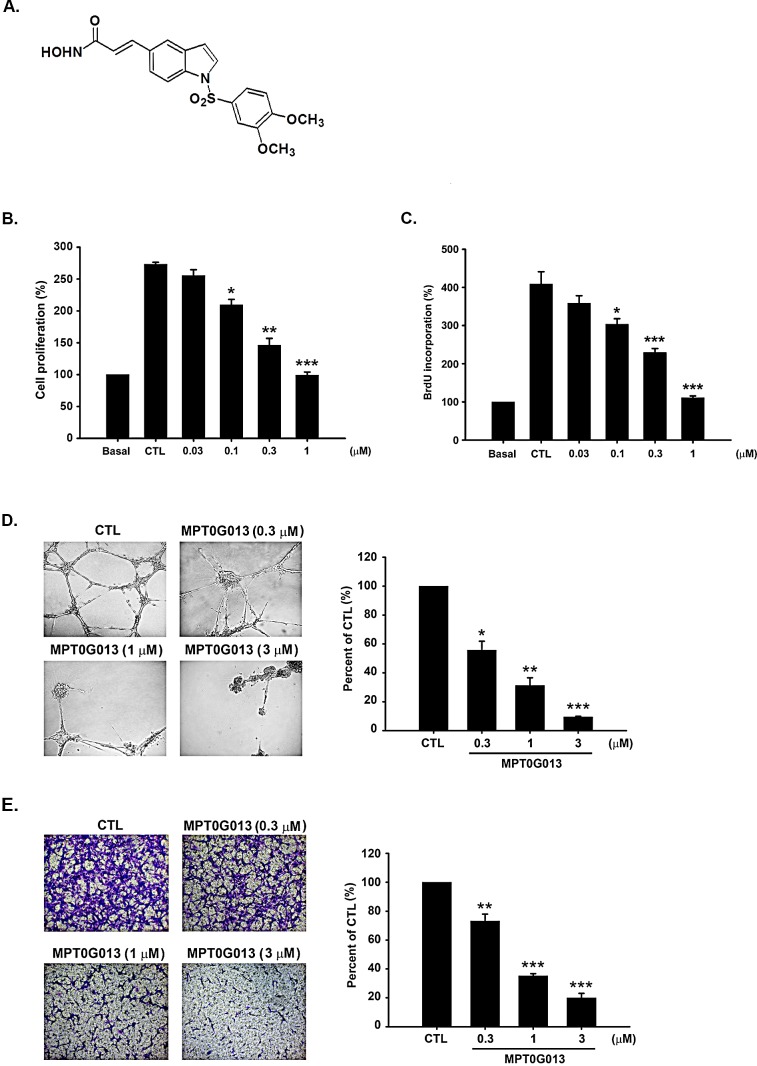
MPT0G013 inhibits angiogenesis *in vitro* A, Chemical structure of MPT0G013. B, HUVECs were treated with or without MPT0G013 at the indicated concentrations in EGM-2 medium. Inhibition of cell proliferation was measured by *crystal violet assay* after 72 hrs. C, DNA synthesis was determined by BrdU incorporation assay. In B and C, 100% = OD. D, *Left panel*, representative photographs of tube formation of HUVECs treated with or without MPT0G013 on matrigel under microscope (magnification is X100). *Right panel*, quantitative analysis of the total tube length by Image analysis software (Image-Pro^®^ Plus). For total tube length 100% = μm. E, *Left panel*, inhibitory effect of MPT0G013 on cell migration using a boyden chamber assay. *Right panel*, quantitative analysis of the migrated cell numbers. 100% = number of migrating cells. Data represent the mean ± SD from three independent experiments. * *P* < 0.05, ** *P* < 0.01 and *** *P* < 0.001 versus control.

Endothelial cell proliferation, tube formation, and migration are essential steps in angiogenesis, and growth factors play important roles during angiogenesis through their mitogenic effects on endothelial cells [2]. Initially, we screened several arylsulfonamide derivatives for antiangiogenic activities using cell-based crystal violet assays. After treatment with or without a series of compounds for 72 hrs in EGM-2 medium, cells were then stained with crystal violet and measured the effect on cell growth. As shown in Table 1 and Figure 1B, MPT0G013 showed the most potent antiproliferative activity, with a GI_50_ value of 0.14 ± 0.01 μM. Therefore, we chose MPT0G013 for further studies of antiangiogenic activities and mechanisms *in vitro* and *in vivo*. To examine the effects of MPT0G013 on angiogenesis, we performed a series of well-established functional assays. Among these, 5-bromo-2-deoxyuridine (BrdU) incorporation assays was carried out in EGM-2 medium. Proliferating HUVECs were starved overnight and then were treated with or without MPT0G013 at various concentrations for total 48 hrs. BrdU-labeling reagent were added at the last 18 h. Figure 1C demonstrated that MPT0G013 inhibits endothelial cell DNA synthesis in a concentration-dependent manner (GI_50_ = 0.19 ± 0.02 μM). Subsequently, we evaluated the effect of MPT0G013 on the formation of capillary networks using Matrigel assays. HUVECs were seeded in a matrigel-coated 96-well plate and treated with or without MPT0G013. Figure 1D shows induction of differentiation by endothelial growth medium-2 (EGM-2; control group) in human umbilical vein epithelial cells (HUVECs), and formation of highly branched networks of capillary-like structures. In contrast, MPT0G013 treatments significantly inhibited tube formation in a concentration-dependent manner. Tube lengths were then analyzed and counted in five randomly selected areas of samples under microscope field using Image analysis software (Image-Pro^®^ Plus). In agreement with the results (Figure 1D, left panel), the right panel in Figure 1D shows 44.3% (*P* < 0.05), 68.8% (*P* < 0.005), and 90.6% (*P* < 0.001) inhibition following treatment with 0.3, 1, and 3 μM MPT0G013, respectively. Because the chemotactic motility of endothelial cells is essential during the angiogenic sprouting process, we used Boyden chamber assays to determine the effects of MPT0G013 on endothelial cell migration. Treatment with MPT0G013 for 6h concentration dependently inhibited EGM-2-induced cell migration (Figure 1E). Taken together, these data indicate that MPT0G013 has potent antiangiogenic activity *in vitro*.

**Table 1:** Anti-proliferative effects of MPT0G013 and several synthetic arylsulfonamide derivative compounds in HUVECs

Compound	GI_>50_ (μM ± SD)
MPT0G013	0.14 ± 0.01
MPT0G017	0.60 ± 0.05
MPT0G018	0.98 ± 0.28
MPT0G019	1.40 ± 0.14
MPT0G031	4.63 ± 0.15
MPT0G032	3.64 ± 0.25
MPT0G033	4.34 ± 0.22
MPT0G034	6.49 ± 0.12
MPT0G036	1.45 ± 0.07
MPT0G037	2.03 ± 0.06
MPT0G038	15.75 ± 0.08
MPT0G039	14.45 ± 0.09
MPT0E014	0.47 ± 0.02
MPT0E028	0.15 ± 0.01
	Mean ± SD (n = 3)

### MPT0G013 induces G0/G1 arrest in HUVECs

**Figure 2:**
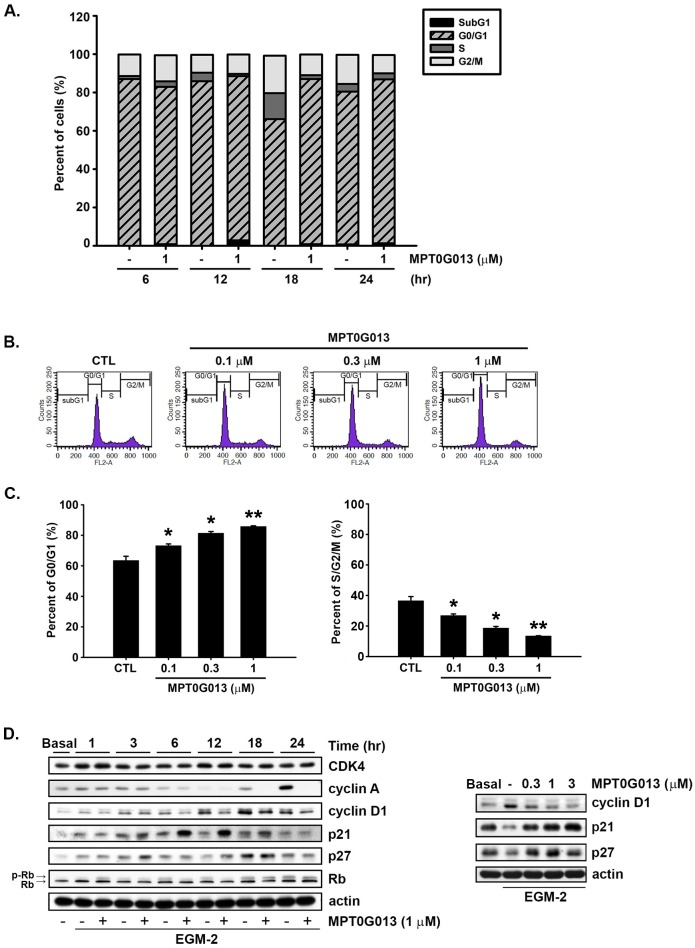
MPT0G013 induces cell cycle arrest in the G0/G1 phase A, After starvation for 24 h, HUVECs were then treated with or without MPT0G013 (1 μM) for the indicated time interval. After labeling with propidium iodide, DNA content was analyzed by flow cytometry. B, HUVECs were treated with or without the indicated concentrations of MPT0G013 for 18 h and were analyzed by flow cytometry for cell cycle distribution. C, Quantification of cell population in G0/G1 and S/G2/M phase. In A, B and C, 100% = percent of cells. D, HUVECs incubated in EGM-2 medium were treated with or without MPT0G013 at indicated times. Cells were harvested and analyzed protein expression by western blot. Basal, starved condition in EBM-2 medium. Data represent the mean ± SD from three independent experiments. * *P* < 0.05 and ** *P* < 0.01 versus control.

To determine whether MPT0G013 impairs cell proliferation, we examined cell cycle phases using flow cytometry assays. In Figure 2A, treatment with MPT0G013 for 18 h increased 20.5% of cells accumulation in the G0/G1 phase and decreased 20.3% of cells in the S/G2/M phase compared with CTL. As shown in Figures 2B–2C, treatment with MPT0G013 increased the percentage of HUVECs in the G0/G1 phase and decreased the population of cells in S, G2, and M phases in a concentration-dependent manner. Subsequently, we examined the effect of MPT0G013 on the expression of cell cycle regulating proteins of the G0/G1 phase. MPT0G013 significantly increased protein expression of p21 (Waf1/Cip1) and p27, and down-regulated the expression of cyclin D1 in a concentration- and time-dependent manner (Figure 2D). Cyclin A and phosphorylated Rb proteins were also down-regulated after 12- and 18-h treatments. Interestingly, MPT0G013 had no effect on the expression of CDK4.

### MPT0G013 inhibits angiogenesis by up-regulating *TIMP3*

To examine the action mechanisms by which MPT0G013 regulates vessel formation *in vitro* and *in vivo*, we initially performed microarray analyses to evaluate expression patterns of angiogenesis-related genes following MPT0G013 treatments in endothelial cells. Supplemental Figure 1 shows clustering analyses of gene expression 24 h after MPT0G013 treatment, and suggests that MPT0G013 up-regulates the antiangiogenic genes *IGFBP3* and *TIMP3*, and down-regulates the pro-angiogenic genes *ANGPT2*, *FGF2*, *Flt1*, and *PLAUR* (Table 2).

**Table 2:** Angiogenic-related genes down-regulated and up-regulated by MPT0G013 in endothelial cells

Accession no.	Gene name	Symbol	Log_2_ ratio	*P* value
NM_001118887.1 *	angiopoietin 2	ANGPT2	−5.070808	1.77579911E-33
NM_003868.1	fibroblast growth factor 16	FGF16	−3.335651	1.2301071E-24
NM_001005377.2 **	plasminogen activator, urokinase receptor	PLAUR	−2.804592	6.2211468E-19
NM_002632.4	placental growth factor	PGF	−2.720818	2.33218952E-14
NM_002006.4	fibroblast growth factor 2 (basic)	FGF2	−2.678275	5.80887784E-16
NM_002658.3 ***	plasminogen activator, urokinase	PLAU	−2.296567	0.000001236864
NM_001160031.1 ^#^	fms-related tyrosine kinase 1 (vascular endothelial growth factor/vascular permeability factor receptor)	FLT1	−1.494546	0.000000085637
NM_000245.2 ^¥^	met proto-oncogene (hepatocyte growth factor receptor)	MET	−1.305731	0.000000299598
NM_001146.3 ^£^	angiopoietin 1	ANGPT1	−1.137254	0.000476530404
NM_001024847.2 ^†^	transforming growth factor, beta receptor II (70/80kDa)	TGFBR2	−1.055459	0.000000567496
NM_203339.1 ^‡^	clusterin	CLU	1.844689	0.000002429692
NM_000362.4	TIMP metallopeptidase inhibitor 3	TIMP3	3.415409	1.19837665E-22
NM_000598.4 ^§^	insulin-like growth factor binding protein 3	IGFBP3	4.038052	3.81784907E-34

HUVECs were treated with 10 μM MPT0G013 for 24 hr, then the total RNA was extracted for microarray analysis. Each unique splice variant of the gene was also tested. Different accession numbers are given.

* ANGPT2: NM_001147.2,NM_001118888.1,

** PLAUR: NM_002659.3.

*** PLAU: NM_001145031.1,

# FLT1: NM_001160030.1,

¥ MET: NM_001127500.1,

£ ANGPT1: NM_001199859.1,

† TGFBR2: NM_003242.5,

‡ CLU: NM_001831.2,NM_001171138.1,

§ IGFBP3: NM_001013398.1

**Figure 3:**
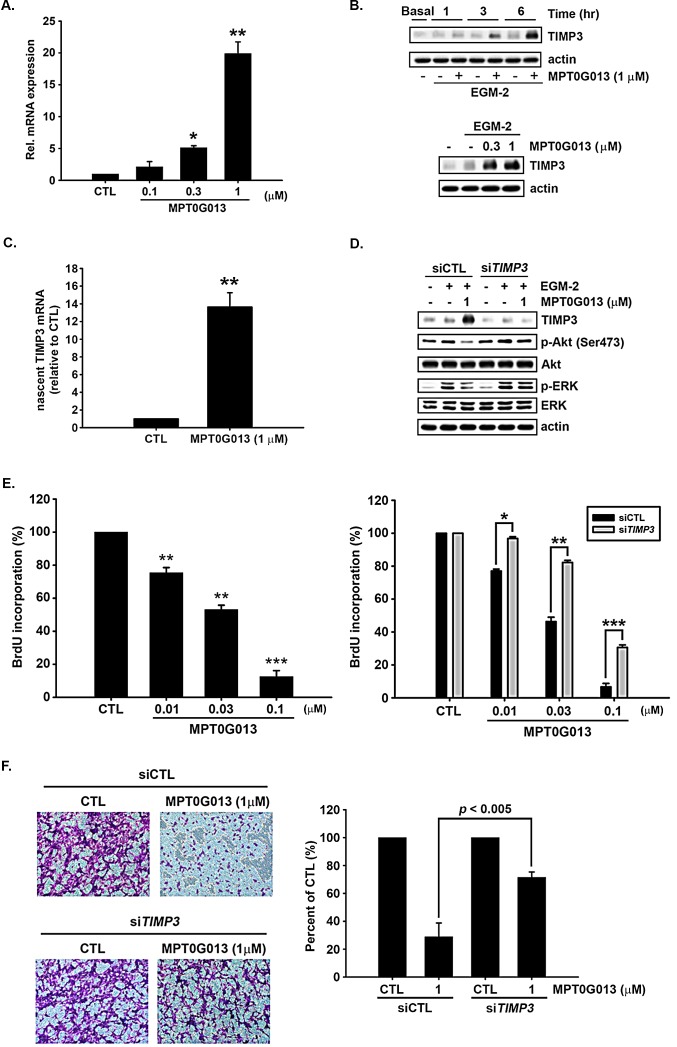
Induction of TIMP3 expression by MPT0G013 inhibits angiogenesis A, Quantitative RT-PCR analysis of TIMP3 mRNA expression in endothelial cells treated with or without MPT0G013 for 6 h. B, *Top panel*, Western blot showing induction of TIMP3 protein expression by MPT0G013 at indicated times. *Bottom panel*, The dose-dependent induction of TIMP3 protein in HUVECs treated with or without MPT0G013 at indicated concentrations for 6 h. C, Nascent RNA was labeled and isolated from HUVECs using Click-iT Nascent RNA Label and Capture Kit (Invitrogen), and then the nascent TIMP3 mRNA was measured using Quantitative RT-PCR. In A and C, 100% = 2^(−CT)^. D, Western blot showing that silencing TIMP3 reversed the inhibition effect of phosphorylated Akt and ERK induced by MPT0G013. E, BrdU incorporation assay. *Left panel*, MPT0G013 dose-dependently inhibited BrdU incorporation after 18 h incubation. *Right panel*, HUVECs tranfected with siTIMP3 increased DNA synthesis after treatment with MPT0G013 for 18 h. F, *Left panel*, boyden chamber chemotaxis assay. Silencing TIMP3 in HUVES rescued the inhibitory effect of MPT0G013 on migration. *Right panel*, quantitative analysis of the migrated cell numbers. Sodium citrate was used to solve crystal violet and then detected absorbance with 550 nm wavelength. In E and F, 100% = OD. Basal, starved condition. Data represent the mean ± SD from three independent experiments. * *P* < 0.05, ** *P* < 0.005 and *** *P* < 0.001 versus control.

To confirm these microarray data, we performed quantitative RT-PCR assays and western blot analyses of *TIMP3* mRNA and protein expression. Figures 3A and 3B show that treatment with MPT0G013 significantly up-regulated *TIMP3* mRNA up to 18-fold, and increased TIMP3 protein expression in a concentration- and time-dependent manner. To further investigate whether MPT0G013 increased TIMP3 expression at the transcriptional or post-transcriptional levels, we utilized the Click-iT^®^ Nascent RNA Capture kit (Invitrogen, Carlsbad, CA, USA) to labeled nascent RNA and isolated from cells. Figure 3C shows that nascent TIMP3 mRNA was significantly up-regulated by MPT0G013 up to 14-fold relative to CTL, indicating that MPT0G013 affected TIMP3 expression at the transcriptional activation. To confirm that TIMP3 is an important mediator of MPT0G013-mediated inhibition of angiogenesis, we knocked down *TIMP3* using specific siRNA (Figure 3D). Figure 3E, *left panel* shows that MPT0G013 inhibited BrdU incorporation at 18 h in a dose-dependent manner. In the *right pane*l of Figure 3E shows rescue of MPT0G013-inhibited DNA synthesis by siTIMP3 after 18-h treatment. Silencing of TIMP3 in endothelial cells also significantly rescued migration in the presence of MPT0G013 (Figure 3F). Previous studies also show that TIMP3 regulates angiogenesis by blocking VEGF signaling through Akt and ERK pathways [15]. Moreover, Figure 3D shows that *TIMP3*-silencing in MPT0G013-treated HUVECs led to a highly significant increase in the expression of phosphorylated Akt and ERK relative to that in the siCTL control group. These observations indicate that MPT0G013 inhibits angiogenesis by up-regulating TIMP3 expression, and subsequently by inhibiting downstream PI3K/Akt and ERK signaling pathways.

### MPT0G013 inhibits tumor angiogenesis and growth by up-regulating TIMP3

**Figure 4:**
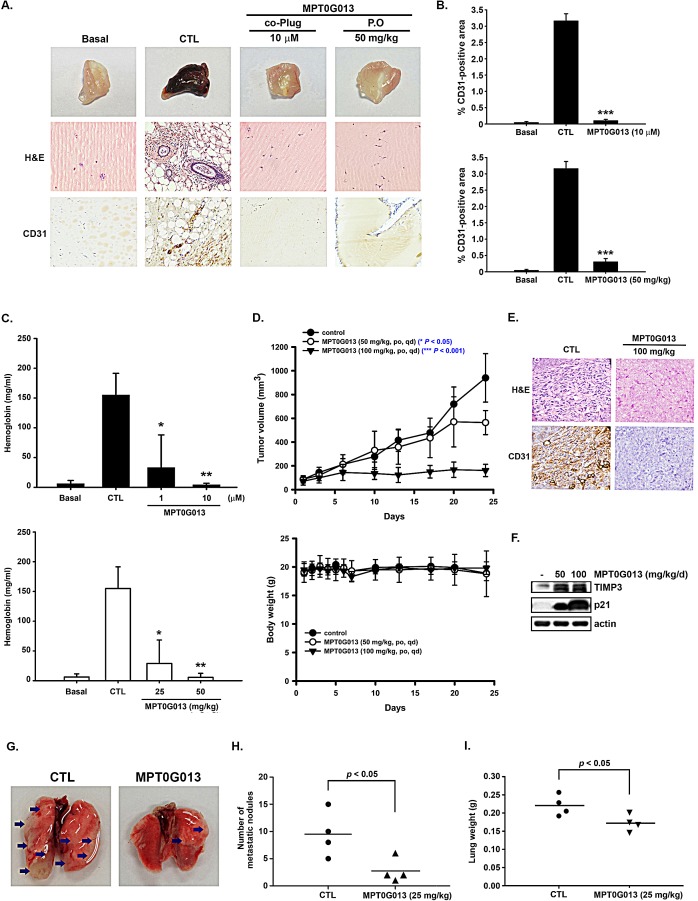
MPT0G013 inhibits the *in vivo* angiogenesis and tumor growth A, Nude mice were injected subcutaneously with matrigel mixed with or without MPT0G013 (1 μM and 10 μM) or oral (p.o.) administration with MPT0G013 (50 mg/kg/d). Plugs were excised from the mice after a week and photographed. Sections of H&E stained Matrigel plugs were examined by light microscopy. The Matrigel plugs were subjected to CD31 immunohistochemical staining. *Brown color*, CD31-positive blood vessels. B, Quantification of CD31-positive area. 100% = pixels. The data are representative of five randomly chosen independent fields using the National Institutes of Health (NIH) Image J software (Bethesda, MD). ***, *p* < 0.001 as compared with the control group. C, Quantification of the hemoglobin content of matrigel plugs by spectrophotometer measured at 540 nm. 100% = OD. Data represent the mean ± SD from five independent experiments. * *P* < 0.05 and ** *P* < 0.005 versus control. D, Effect of MPT0G013 on the growth of HCT116 colon tumor xenografts in BALB/c nude mice. *Top panel*, Tumor growth is presented as the mean tumor volume (mm^3^) ± S.E. Tumor volume was determined by caliper measurements and was calculated as the product of 1⁄2 × length × width^2^. *Bottom panel*, body weight (g) of the mice. Each value represents the mean of at least five animals. ***, *p* < 0.001 as compared with the control group. E, CD31-stained sections of blood vessels from a xenograft tumor. *Brown color*, CD31-positive blood vessels. F, Western blot analysis of TIMP3 and p21 expression in tumor tissue. G, MPT0G013 reduced colon cancer cell metastasis *in vivo*. Proliferating HCT-116 cells were injected into the lateral tail vein of SCID mice that received vehicle or MPT0G013 (25mg/kg) by oral administration every other day (n = 4 in each group). Representative images of metastatic lung nodules from mice after treated with or without MPT0G013 for 6 weeks. Arrows indicate surface lung nodules. H, Macroscopic lung surface nodules (Ф > 1 mm) were counted under a dissected microscope (Optima) in each group. Black bar represent the average. I, Quantification of gross weight of individual lungs.

To investigate the effects of MPT0G013 on angiogenic growth factors *in vivo*, we performed Matrigel plug assays. Growth factors were mixed with Matrigel and were implanted subcutaneously into *nu/nu* mice. After 7 days, the Matrigel plugs were excised following hematoxylin and eosin (H&E) staining and immunohistochemical staining for the angiogenic marker CD31. The CD31-postive area was quantified by using the NIH Image J software (Bethesda, MD). In these experiments, significant antiangiogenic effects were observed after oral administration of MPT0G013 (Figure 4A and 4B). Subsequent hematoxylin and eosin (H&E) staining and CD31 staining also showed decreased vasculature in MPT0G013 infused gels than in growth factor-only infused control plugs (Figure 4A and 4B). Angiogenesis was then quantified by measuring hemoglobin contents in plugs, which showed that MPT0G013 significantly inhibited angiogenesis by 96% at 10 μM. Furthermore, MPT0G013 also showed significant antiangiogenic effect by 84% and 97% after oral administration at doses of 25 and 50 mg/kg/d, respectively. (Figure 4C).

Numerous studies demonstrate that tumor growth is dependent on angiogenesis and that inhibition of angiogenesis can attenuate tumor growth. In the present study, we further evaluated whether MPT0G013 directly affects tumor growth and angiogenesis in a colon carcinoma (HCT116) xenograft model. These experiments showed no symptoms of toxicity such as weight loss in MPT0G013-treated mice (n = 5; Figure 4D, *top panel*), indicating tolerance of treatments. Untreated control HCT116 xenografts grew to >900 mm^3^ in 25 days, whereas the mean tumor volumes in MPT0G013 treated mice (50 and 100 mg/kg/day) were <550 mm^3^ and <160 mm^3^ at this time point, respectively (Figure 4D, *bottom panel*). To further investigate inhibition of tumor growth and angiogenesis by MPT0G013, we performed immunohistochemical staining for the angiogenic marker CD31. Administration of MPT0G013 significantly decreased the numbers of CD31-expressing vessels relative to those in the CTL group (Figure 4E). Moreover, these *in vivo* antiangiogenic and antitumor effects were corroborated in western blot analyses of drug-induced intratumoral markers. Figure 4F shows that the significant increases in protein expression of TIMP3 in tumor tissues from the MPT0G013-treated mice were accompanied by up-regulation of p21. Although angiogenesis is essential for tumor growth and metastasis and constitutes an important role in the control of cancer progression [17], previous studies showed that anti-angiogenic therapies may accelerate invasion and metastasis. Sunitinib, a small-molecule receptor tyrosine kinase inhibitor and the anti-VEGFR2 antibody DC101 stimulated tumor metastasis despite their inhibition of primary tumor growth in some cases [18, 19]. We therefore generated lung tumor metastases model to evaluate the effect of MPT0G013 treatment on cancer cell metastasis *in vivo*. HCT-116 colon cancer cells were injected into the tail vein of severe combined immunodeﬁciency (SCID) mice, which then received vehicle treatment or MPT0G013 (25mg/kg every other day) just after tumor cells inoculation. At the study endpoint, the lungs in mice were surgically resected and evaluated. Compared to the control group, the mice treated with 25 mg/kg MPT0G013 exhibited significantly decreased levels and numbers of lung surface metastasis (Figure 4G, 4H). The lung weights were also reduced in the MPT0G013 treatment group relative to the control group (Figure 4I). The results demonstrate that MPT0G013 does not promote metastasis in the HCT-116 metastasis animal model, instead, may interrupt the cancer cells metastasis *in vivo*. Taken together, these results indicate that MPT0G013 abrogates growth factor-induced angiogenesis by up-regulating the antiangiogenic protein TIMP3 *in vitro* and *in vivo*, and leads to tumor growth inhibition.

## DISCUSSION

TIMP3 was originally identified as an *in vitro* and *in vivo* inhibitor of angiogenesis in 1997 [10], and for many years, it was accepted that TIMPs primarily inhibit MMPs [20, 21]. However, more recent evidence indicates multiple functions of TIMPs that are independent of MMP-inhibitory properties. Furthermore, inhibition of angiogenesis by TIMP3 was suggested to be distinct from MMP-inhibitory activities, and recent evidences indicate that inhibition of VEGF-induced cell proliferation and migration by TIMP3 reflects decreased binding of VEGF to VEGFR2 [15]. Loss of *TIMP3* has been shown to enhance hepatoprotective EGFR ligand shedding and downstream ERK1/2 phosphorylation in mouse embryonic fibroblasts (MEFs) [22]. Moreover, lentiviral overexpression of TIMP3 in mensenchymal cells led to decreased phosphorylation of ERK and Akt [23]. In addition, accumulating evidence indicates that *TIMP3* is frequently down-regulated in various types of cancer [11, 24]. Furthermore, numerous studies have demonstrated that activation of PI3K/Akt and Ras/Raf/ERK signaling by many growth factors is essential for normal cellular G1/S phase transitions, cell proliferation, and migration of endothelial cells [25-27]. Data from preclinical studies demonstrate that inhibitors of PI3K/Akt and ERK signaling pathway are potent antiangiogenic agents that inhibit tumor growth [28, 29]. In the present study, silencing of TIMP3 significantly reversed the inhibitory effects of MPT0G013 on Akt and ERK phosphorylation, suggesting that TIMP3 is an important target of MPT0G013. Although the data in Table 2 show down-regulation of angiogenic genes by MPT0G013, siTIMP3 significantly rescued DNA synthesis and cell migration in MPT0G013-treated HUVECs, suggesting that MPT0G013-induced TIMP3 plays a major role in the observed antiangiogenic effects. However, MPT0G013 also inhibited *ANGPT2*, *FGF16*, *PLAUR* and *PlGF* gene expression (Table 2 and Supplemental Figure 2). Therefore, down-regulation of angiogenic genes may also contribute to the antiangiogenic effect of MPT0G013. We also observed only slight decreases in MMP-2 and MMP-9 protein expression after treatment with MPT0G013 (data not shown). However, because silencing of TIMP3 enhanced the migration of endothelial cells through gelatin-coated membranes (both-sided), the contribution of targeting these MMPs may only represent a minor effect of MPT0G013.

Microarray analyses showed that MPT0G013 inhibited the expression of various angiogenic genes and up-regulated the anti-angiogenic genes, including *IGFBP3* and *TIMP3*. The role of insulin-like growth factor binding-protein 3 (IGFBP3) in inhibition of VEGF-induced endothelial cell growth and antiangiogenesis is well established [30, 31]. Thus, we confirmed the effects of MPT0G013 on IGFBP3 mRNA expression using quantitative RT-PCR, and showed 7-fold induction relative to the CTL (Supplemental Figure 3A). However, in BrdU incorporation assays, silencing of IGFBP3 in MPT0G013-treated cells only slightly reversed the antiproliferative effects relative to the CTL group (Supplemental Figure 3B). Therefore, we assumed that IGFBP3 may also contribute to the antiangiogenic activities of MPT0G013 but plays relatively a minor role compared with TIMP3.

MPT0G013 is reportedly a novel HDAC inhibitor that suppresses tumor cell growth [16]. We confirmed more potent inhibition of HDAC activity by MPT0G013 than by the HDAC inhibitor SAHA (Supplemental Figure 4 and Supplemental Table 1). Previous studies showed that SAHA was found to be a potent inhibitor of class I isoforms (HDAC1, 0.03 μM; HDAC2, 0.92 μM) and class IIb (HDAC6, 0.03 μM) in biochemical assays, with relatively weak inhibitory activity for the class IIa isoforms [32]. We showed that SAHA inhibited HDAC1, HDAC2, and HDAC6 with IC_50_ of 118.4, 506.5 and 113.5 nM, respectively (Supplementary Table 1). The IC_50_ values from our results represented the similar pattern of HDAC inhibitory effect of SAHA compared with previous studies. Evidence also demonstrated that utilizing the same kit (HDAC Biochemical Assay kit, BPS Biosciences, CA, USA) showed significantly different IC_50_ values [33-35]. We assumed that the activities from different batches of enzymes are various, and also affected by its laboratory environmental conditions. However, our results showed that MPT0G013 and SAHA were Class I/IIb HDAC inhibitors and MPT0G013 is more potent than SAHA. Moreover, MPT0G013 significantly induced TIMP3 mRNA and protein expression in endothelial cells and tumor xenografts, and whereas SAHA had a similar pattern of inhibitory activities on HDAC subtypes, it barely induced TIMP3 gene or protein expression *in vitro* (Supplemental Figure 5A) or *in vivo* (Supplemental Figure 5B and 5C). Hence, we suggest that this effect is an exclusive novel property of MPT0G013.

We evaluated the anti-proliferative effect of MPT0G013 on various human cancer cell lines using SRB assay. Supplemental table 2 showed that MPT0G013 inhibited the proliferation of A549, HCT-116, Hep3B, MDA-MB-231, PC-3 and SK-OV-3 with GI_50_ values of 0.44, 0.34, 0.57, 0.32, 0.42, 0.35 μM. The GI_50_ values of MPT0G013 in cancer cell lines are about 3-fold higher compared to MPT0G013 in HUVECs. From the results, we could not rule out the effect of MPT0G013 on tumor cells in the xenograft model. However, MPT0G013 significantly inhibited growth factors-induced blood vessel formation in the matrigel plug assay and the tumor xenograft model. Therefore, we suggest that MPT0G013 is a potent anti-angiogenic agent *in vivo*.

In conclusion, to the best of our knowledge, this is the first report to show that the novel antiangiogenic agent MPT0G013 up-regulates the tumor suppressor gene TIMP3 in endothelial cells, and subsequently attenuates downstream signaling via PI3K/Akt and ERK pathways. Using Matrigel plug assays and the tumor xenograft models, we provide strong evidence that MPT0G013 inhibits tumor angiogenesis *in vivo*. Furthermore, *ex vivo* immunoblot analyses showed significant up-regulation of p21 and TIMP3 expression in tumors from MPT0G013-treated mice. In summary, the present data demonstrate MPT0G013 as a novel candidate for the treatment of angiogenesis-related diseases such as cancer.

## MATERIALS AND METHODS

### Reagents

MPT0G013 was synthesized by Professor Jing-Ping Liou's Lab. (School of Pharmacy, College of Pharmacy, Taipei Medical University, Taiwan), and the purity is more than 99%. Medium 199, fetal bovine serum (FBS), penicillin, streptomycin and other tissue culture reagents were obtained from Gibco BRL Life Technologies (Grand Island, NY). Endothelial cell basal medium (EBM) and endothelial growth factors (EGM-2) were purchased from Clonetics (BioWhittaker, Walkersville, MD). Propidium iodide was obtained from Sigma Chemical (St. Louis, MO). Matrigel basement membrane matrix were purchased from BD Biosciences (San Jose, CA). TRIzol reagent was from Invitrogen (Carlsbad, CA), random primer and M-MLV RT were purchased from Promega (Madison, WI). The antibody against p21, TIMP3, p-ERK, p-Akt (Ser473), ERK, and Akt were purchased from Cell Signaling Technology (Beverly, MA). The antibody against cyclin D was purchased from Calbiochem (San Diego, CA). Antibodies against p27, CDK4, cyclin A, and Rb were purchased from Santa Cruz Biotechnology (Santa Cruz, CA). Actin antibody was purchased from Chemicon (Billerica, MA).

### Cell culture

Human umbilical vein endothelial cells (HUVECs) (BCRC, H-UV001) was purchased from Food Industry Research and Development Institute, Hsin Chu, Taiwan and were grown to confluence on 1% collagen, and maintained in endothelial cell medium (ECM) (ScienCell Research Laboratory, Carlsbad, CA) supplemented with 20% FBS, 1:5 Penicillin/Streptomycin (ScienCell Research Laboratory, Carlsbad, CA) and 1:5 endothelial cell growth supplements (ECGs) (ECGs; Upstate Biotechnology Inc., Lake Placid, NY). Before treatment of MPT0G013 in EGM-2 medium, HUVECs were starved for 24 hours in EBM-2 medium. All of the following experiments were performed in this medium. And basal represented the starvation condition.

### *In vivo* matrigel plug assay

Female nude-athymic mice of 6 weeks old and weighing ~20g were used in matrigel plug assay, and all animal experiments followed ethical standards, and protocols have been reviewed and approved by Animal Use and Management Committee of National Taiwan University (NTU IACUC approved No. 20120533). Mice were divided into two groups with 3 animals in each group. One experiment was injected abdomens subcutaneously with Matrigel (BD Bioscience) mixed with several angiogenic factors (40 ng/ml), with or without MPT0G013 (1 μM and 10 μM), while the other experiment was injected with Matrigel containing growth factors only and subsequently given MPT0G013 (25 mg/kg/d and 50 mg/kg/d) by oral administration. After seven days, the animals were sacrificed and the matrigels were carefully dissected and photographed. To quantify the blood vessel formation, hemoglobin content was analyzed by Drabkin's reagent kit (Sigma Chemical, St. Louis, MO).

### *In vivo* mouse tumor xenograft assay

Male 4 weeks old BALB/c nude mice were implanted s.c. with HCT-116 cells. When the tumors reached the average volume of 90 mm^3^, the mice were divided into four groups (n = 5) and then were treated orally with vehicle (1% carboxymethyl cellulose + 0.5% Tween 80, 0.2 mL/mouse) or MPT0G013 (50 and 100 mg/kg/d). The length (L) and width (W) of the tumor were measured every 3 to 4 days, and the tumor volume was calculated as *LW*^2^ / 2. The protocols of the *in vivo* study were approved by the Animal Care and Use Committee at National Taiwan University.

### *In vivo* metastatic animal model

In the metastatic experiment, HCT-116 colon cancer cells (1 × 10^7^ cells/ml) were injected directly into the lateral tail vein of 4 weeks old male SCID mice. After the intravenous tumor inoculation, vehicle (1% carboxymethyl cellulose + 0.5% Tween 80, 0.2 mL/mouse) or MPT0G013 (25mg/kg) was given by oral administration every other day (n = 4 in each group). Mice were sacrificed at 42 days, and the lungs were removed, photographed and fixed in 4% formaldehyde. The lung surface metastatic nodules (Ф > 1 mm) were counted under the dissected microscope (Optima) and the gross weight of individual lungs were quantified. The protocols of the *in vivo* study were approved by the Animal Care and Use Committee at National Taiwan University.

### Crystal violet assay

Growing HUVECs seeded in 96-well culture plates (5.0 × 10^3^ cells per well) were treated with MPT0G013 in EGM-2 medium at various concentrations (0.01-1 and 0.1-10, respectively). After 3 days of incubation at 37°C, the cells were stained with 0.1% crystal violet for 10 minutes. Then the dye was eluted by 0.1 M sodium citrate, and absorbance is measured at 540 nm with an ELISA reader. The relative percent of cell growth was calculated by comparison between the control and the MPT0G013-treated wells.

### DNA synthesis assay

Cell proliferation was measured by 5-bromo-2-deoxyuridine (BrdU) incorporation into newly synthesized DNA of growing HUVECs with the colorimetric ELISA assay (Chemicon, Temecula, CA). Endothelial cells seeded in 96-well culture plates (5.0 × 10^3^ cells per well) were starved with EBM-2 medium overnight. Then the cells were treated with EGM-2 medium alone (control) or medium with different concentrations of MPT0G013 for 48 hours or indicated times. Growth factor-containing EGM-2 medium prompted the cells to re-enter the cell cycle, allowing us to monitor the cells movement into S phase and G2. To label the synthesized DNA, 10 μM BrdU was added to cultures for the last 18 hours before staining. After incubation, the cells were fixed and detected by BrdU ELISA according to the manufacturer's instructions.

### Boyden Chamber migration assay

The evaluation of HUVEC cell migration was performed by using boyden chamber with 8 μM pore sized filters which was pre-coated with 0.5 % gelatin both sides. Proliferating HUVECs were pretreated with the indicated concentrations of MPT0G013 for 6h, and then the cells were harvested and resuspended in serum-free EBM-2 media with the indicated concentrations of MPT0G013. An equal number of cells (10^5^) were seeded to the chambers in triplicate. To allow for cell migration, EGM-2 medium was added to the lower chamber as chemoattractant. After six hours incubation, the membranes containing migrated cells were fixed and stained with 0.1% crystal violet, and the cells on the upper filter were removed with the cotton swabs. The migrated cells were counted and photographed under a microscope. Five fields were randomly chosen for each membrane, and the results were expressed as percent of migrated cells.

### Capillary tube formation assay

HUVECs were cultured in complete EGM-2 medium with the indicated concentrations of MPT0G013 and seeded onto the Matrigel (Growth factor reduced, BD Biosciences) which was coated in the 96 wells at density of 1.75 × 10^4^ cells/well. After 18 hours of incubation, tubular network structures were visualized and photographed using a phase contrast microscope. The relative tube lengths were measured and analyzed by using Image analysis software (Image-Pro^®^ Plus).

### Flow cytometric analysis

Briefly, HUVECs were starved with EBM-2 medium overnight and were subsequently replaced with EGM-2 medium with or without MPT0G013 (0.1, 0.3, 1 μM) at the indicated times. After trypsinized and fixed in ice-cold 75% methanol for one hour at −20°C, HUVECs were washed with PBS and resuspended in 0.2 ml DNA extraction buffer (0.2 M Na_2_HPO_4_, 0.1 M citric acid; pH 7.8) for 30 minutes. Then the cells were stained with propidium iodide solution (PI; 100 μg/ml RNase, 80 μg/ml propidium iodide, 0.1% Triton X-100) in PBS. FACScan flow cytometry was utilized to determine Cell cycle distribution, and data analysis was performed with CellQuest software (BD Biosciences).

### Western blotting

HUVECs were harvested after treatment of MPT0G013 in EGM-2 medium, and the total cell lysates were prepared in a modified RIPA buffer (150 mM NaCl, 1 mM EDTA, 1% Nonidet p-40, 0.5% sodium deoxycholate, 0.1% SDS, 20 mM Tris, pH 8.0) with protease inhibitors (1 μg/ml aprotinin, 1 μg/ml leupeptin, 0.5 M NaF, 0.5 M Na_3_VO_4_, 1 mM phenylmethylsulfonyl fluoride). The cellular proteins were applied to immunoblot separated by 8%-12% poly-acrylamide gel electrophoresis followed by electroblotting onto polyvinylidene difluoride membranes. Membranes were then blocked with 5% nonfat milk, washed with PBS, probed with specific antibodies and measured by utilizing an enhanced chemiluminescence (ECL) detection system.

### Microarray analysis

HUVECs were treated with or without MPT0G013 for 24 hours and total RNAs were harvested by TRIzol reagent. Samples were processed according to the procedure of Human OneArray from Phalanx Biotech (Hsinchu). The data were analyzed by the Rosetta Resolver System (Rosetta Biosoftware, Seattle, WA)., The fold change of gene expression is duplicated and established at |log_2_ ratio| n≥ 1 and *P* < 0.05, by using DAVID Bioinformatic Resources (NIH, Bethesda, MD) subjected to gene ontology analysis compared with the control sample. Microarray data is has been uploaded to the Gene Expression Omnibus (http://www.ncbi.nlm.nih.gov/geo; accession number GSE56927). The data is private until April 20, 2017.

### RNA isolation, cDNA synthesis, and quantitative reverse transcription PCR

Total RNA was isolated with TRIzol reagent by a standard protocol. mRNA (5 μg) was incubated with random primer at 65°C for 5 minutes, and then reacted with M-MLV RT at 37°C for 1 hour to obtain cDNA. Quantitative PCR was performed with mastermix (TaqMan One Step RT-PCR; ABI) in a total reaction volume of 20 μl per reaction, containing 10 ml of SYBR green PCR master mix (Applied Biosystems), 5 pmol of each forward and reverse primer and 2 μl of cDNA. Amplification of TIMP3 was performed using the following primers: 5′-TGCTCTCTGTCTCTTTTTTCAGCTT-3′ (forward) and 5′-CTACAGTGTGTTGTCTGCTGCTTTT-3′ (reverse). GAPDH was used as an endogenous control.

### Label and capture nascent RNA

Newly synthesized RNA was isolated using Click-iT Nascent RNA Capture Kit (Invitrogen, Carlsbad, CA, USA) according to the manufacturer's instruction. Briefly, HUVECs were treated with or without MPT0G013 (1 μM) and incubated in 0.2 mM of 5-ethymyluridine (EU, an alkyne-modified uridine analog, which is efficiently and naturally incorporated into the nascent RNA) for total 6 h and total RNA was isolated using TRIzol reagent. Then, a click reaction was performed using 5 μg of EU-labeled RNA and 0.5 mM biotin azide; the mixture was incubated at room temperature for 30 min. Following RNA precipitation, the RNA was resuspended in 30 μl of RNase-free water. Biotin-labeled EU-RNA-binding pull-down assay was performed using 12 μl of Dynabeads○RR MyOne^TM^ Streptavidin and bound RNA was washed. The cDNA synthesis was performed directly on the beads using Superscript VILO cDNA synthesis kit (Invitrogen) followed by analysis with QRT-PCR.

### Statistical analysis

The significance of differences *in vivo* data was analyzed by the Mann-Whitney U test. Tumor growth is presented as the mean tumor volume (mm^3^) ± S.E; others represent the mean ± SD of at least three independent experiments. Statistical analysis was performed by the *t*-test, and *P* values less than 0.05 (* *P* < 0.05, ** *P* < 0.01, *** *P* < 0.001) were considered significant.

## SUPPLEMENTARY MATERIAL METHODS, FIGURES, TABLES


