# Supplementary material for: A novel action mechanism for MPT0G013, a derivative of arylsulfonamide, inhibits tumor angiogenesis through up-regulation of TIMP3 expression
Source: Oncotarget. 2014 Sep 8;5(20):9838–50. doi: 10.18632/oncotarget.2451 (PMC4259441; doi:10.18632/oncotarget.2451)
Supplement: Supplementary file 1 [file oncotarget-05-9838-s001.pdf]

A novel action mechanism for MPT0G013, a derivative of arylsulfonamide, inhibits tumor angiogenesis through up-regulation of TIMP3 expression

Supplementary Material

Supplemental Methods

Sulforhodamine B Assay

A549, HCT116, Hep3B, PC-3 and SKOV-3 cells were seeded in 96-well plates in medium with 5% fetal bovine serum overnight. Cells were fixed with 10% trichloroacetic representing cell population at the time of drug treatment (T0). After incubation with vehicle (0.1% DMSO), MPT0G013 SAHA for 48 h, cells were fixed with 10% trichloroacetic acid and then stained with sulforhodamine B at 0.4% (w/v) in 1% acetic acid. Excess Sulforhodamine B was washed away by 1% acetic acid and dye-containing cells were lysed with 10 mmol/L Trizma base. The absorbance was read under wavelength of 515 nm. By measuring time zero (T0), control growth (C), and cell growth in the presence of the drug (Tx), the percentage growth was calculated. Percentage growth inhibition was calculated as 100-[(Tx–T0)/(C-T0)]6100. Growth inhibition of 50% (GI50) is determined at the drug concentration that results in 50% reduction of total protein increase in control cells during the compound incubation.

HeLa nuclear extract HDAC activity assay

HDAC assay with HeLa nuclear extract was carried out by using the HDAC Fluorescent Activity Assay Kit (BioVision, CA, USA) according to manufacturer's instructions. Nuclear extracts from HeLa cells and the HDAC fluorometric substrate were suspended in the assay buffer and then incubated with or without MPT0G013 or SAHA at the indicated concentrations in a 96-well plate at 37°C for 30 min. The reaction was stopped by adding lysine developer, and the mixture was incubated for another 30 min at 37°C. To determine HDAC activity, A fluorescence plate reader with excitation at 355 nm and emission at 460 nm was utilized. And the relative percent of HDAC activity was calculated by comparison between the control and the MPT0G013-treated wells.

Pan-HDACs enzymatic activity assay

Pan HDAC activity was measured by Boc-Lys(Ac)-AMC fluorometric HDAC activity assay kit (BioVision, Mountain View, CA, USA). HUVECs cells were treated

with MPT0G013 or SAHA for 24 h, and then Cells were collected and the nuclear extract proteins were analyzed by utilizing a Fluorometric HDAC Activity Assay Kit (k330-100; BioVision). A fluorescence plate reader with excitation at 355 nm and emission at 460 nm was used to quantify HDAC activity. And the relative percent of HDAC activity was calculated by comparison between the control and the MPT0G013-treated wells.

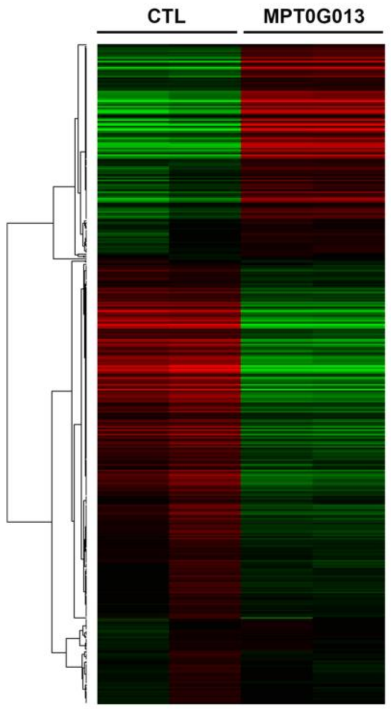

Supplemental Figure 1.
Hierarchical clustering analysis of microarray data comparing MPT0G013-treated group and untreated in HUVECs. Total RNA of HUVECs treated with or without 10 μM MPT0G013 for 24 hours was extracted and analyzed by Human OneArray. Genes significantly different, with $P < 0.05$ after the treatment, were pooled and used to generate heat maps. Up-regulated and downregulated genes are represented in red and green, respectively.

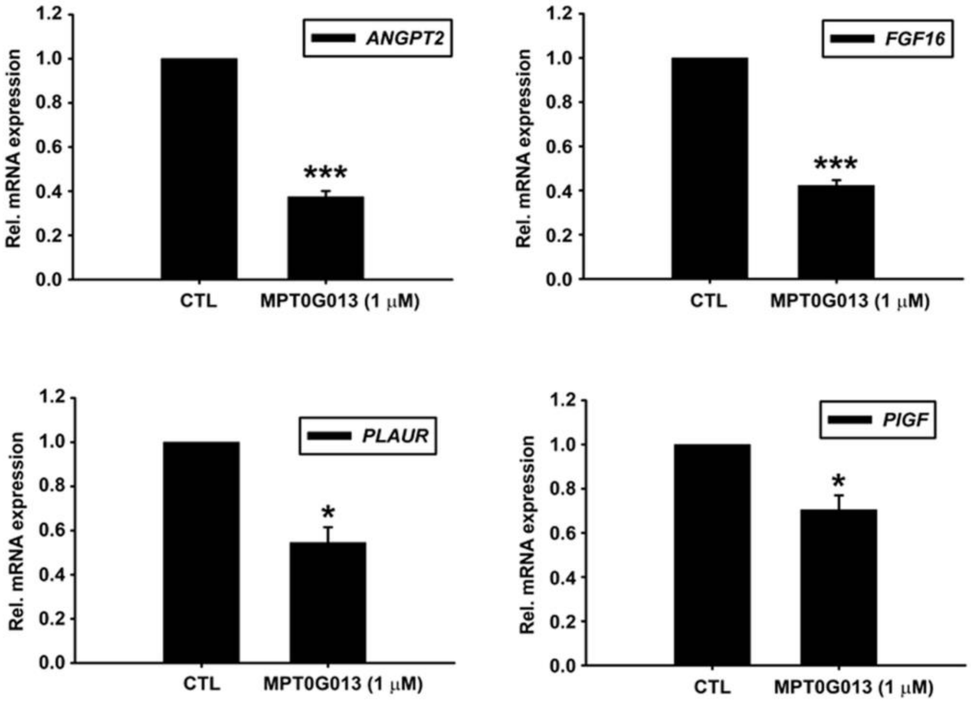

Supplemental Figure 2

Quantitative RT-PCR analysis of indicative mRNA expression in endothelial cells treated with or without MPT0G013 for 24 h. Data represent the mean ± SD from three independent experiments. *p < 0.05 and ***p < 0.001 versus control.

A.

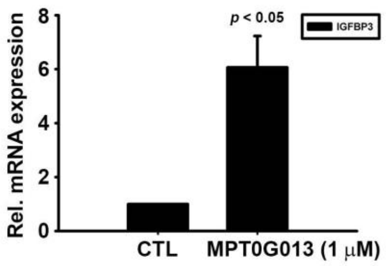

B.

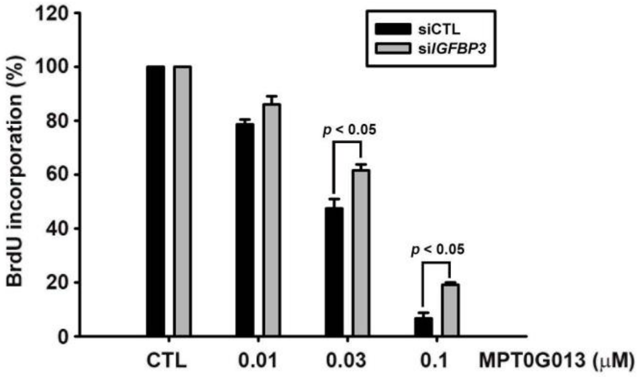

Supplemental Figure 3

A, Quantitative RT-PCR analysis of IGFBP3 mRNA expression in endothelial cells treated with or without MPT0G013 for 12 hr. B, BrdU incorporation assay. HUVECs tranfected with siIGFBP3 slightly increased DNA synthesis after treated with MPT0G013 for 18 hr.

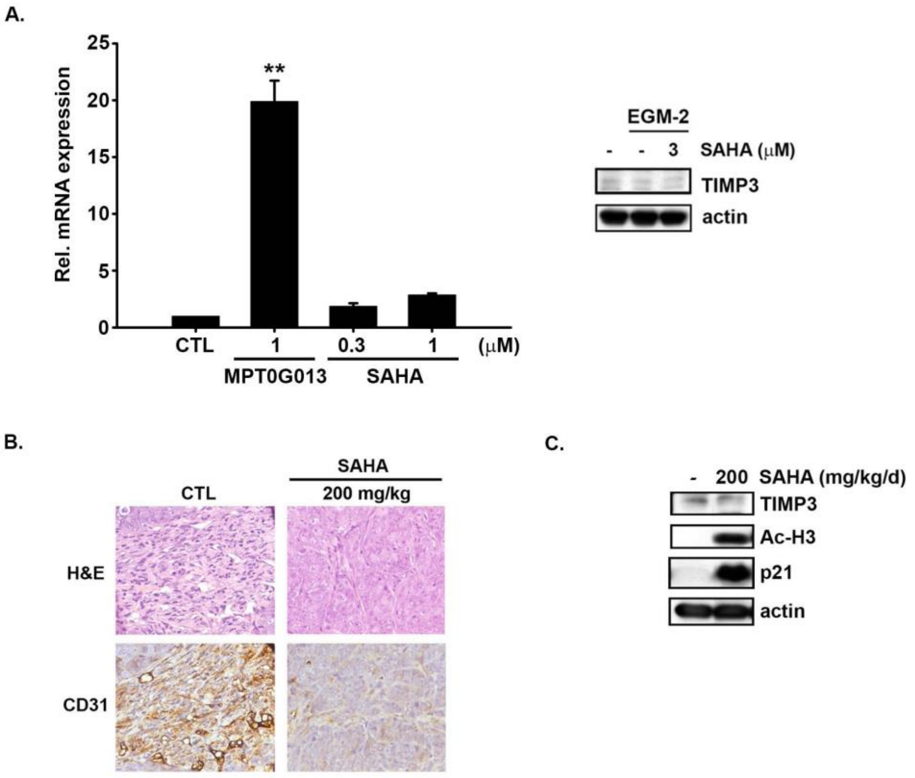

Supplemental Figure 5

A, Left Panel, quantitative RT-PCR analysis of TIMP3 mRNA expression in endothelial cells treated with or without MPT0G013 and SAHA for 6 hr. Right panel, Western blot showing induction of TIMP3 protein expression by SAHA at indicated concentration. B, CD31-stained sections of blood vessels from a xenograft tumor. Brown color, CD31-positive blood vessels. C, Western blot analysis of TIMP3, Ac-H3 and p21 expression in tumor tissue.

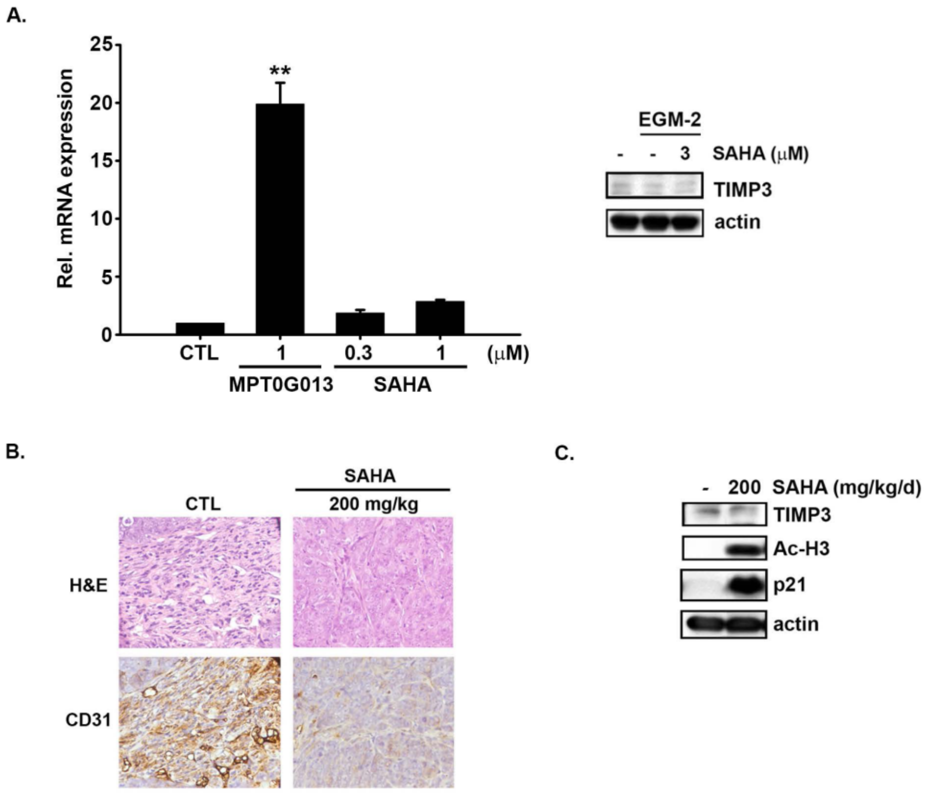

Supplemental Figure 5

A, Left Panel, quantitative RT-PCR analysis of TIMP3 mRNA expression in endothelial cells treated with or without MPT0G013 and SAHA for 6 hr. Right panel, Western blot showing induction of TIMP3 protein expression by SAHA at indicated concentration. B, CD31-stained sections of blood vessels from a xenograft tumor. Brown color, CD31-positive blood vessels. C, Western blot analysis of TIMP3, Ac-H3 and p21 expression in tumor tissue.

Supplemental Table 1. Inhibitory effect on HDAC activity by MPT0G013 and SAHA.

Compound	HDAC1	HDAC2	HDAC8	HDAC4	HDAC6
	Class I			Class IIa	Class IIb
	IC_{50} (nM ± SD)				
MPT0G013	56.7 ± 2.5	125.9 ± 5.1	3322.8 ± 480.5	>10000	56.5 ± 2.6
SAHA	118.4 ± 11.1	506.5 ± 35.6	>10000	>10000	113.5 ± 2.8

Supplemental Table 2. Anti-proliferative effects of MPT0G013 in various cancer cell lines.

Cell line	GI_{50} (μM ± SD)
A549	0.44 ± 0.029
HCT-116	0.34 ± 0.027
Hep3B	0.57 ± 0.049
MDA-MB-231	0.32 ± 0.014
PC-3	0.42 ± 0.014
SK-OV-3	0.35 ± 0.019

Mean ± SD (n = 3)
